# Editorial: Understanding plant cell wall recalcitrance for efficient lignocellulose processing

**DOI:** 10.3389/fpls.2023.1284658

**Published:** 2023-10-03

**Authors:** Holger Klose, Gabriel Paës

**Affiliations:** ^1^ Institute of Bio- and Geosciences, IBG-2: Plant Sciences, Forschungszentrum Jülich, Jülich, Germany; ^2^ RWTH Aachen University, Aachen, Germany; ^3^ Université de Reims Champagne-Ardenne, INRAE, FARE, UMR A 614, Reims, France

**Keywords:** cell wall, recalcitrance, biofuel, biomass processing, biomass

Plant cell walls are complex structures fulfilling a variety of functions throughout a plant’s life. They maintain the plant’s structural integrity by resisting hydrostatic pressure, and at the same time, provide flexibility to support cell growth and division while being an environmental barrier that defends against biotic and abiotic stress. Cell walls are therefore difficult to degrade, which is a major obstacle for many approaches to utilise plant material (McCann and Carpita, 2015; Zoghlami and Paës, 2019). An enhanced understanding of the complex interplay of the different cell wall compounds and their contribution to the cell wall´s resistance towards conversion reactions will lead to more sophisticated strategies to utilize plant biomass for materials, chemicals, and fuels.

This resistance of plant cell walls to degradation or conversion can be described by the concept of biomass recalcitrance. This concept describes the global resistance towards any catalytic attack by mechanical, physical, chemical, or biological forces (McCann and Carpita, 2015). It is due to the complex structure and composition of cell walls, which are made up of different types of polymers such as cellulose, hemicelluloses, and lignin that are tightly interconnected.

Biomass recalcitrance poses a major challenge to the development of sustainable biorefineries and the production of renewable energy and bio-based products. To overcome this challenge, researchers are exploring various strategies to improve the efficiency and effectiveness of biomass conversion processes, such as pretreatment methods that can weaken the cell wall structure and improve the accessibility of enzymes to the plant cell wall components.

This Research Topic consists of 7 original research articles which addressed different aspects of generating a better understanding of the chemical composition and structure of plant cell walls and their relation to cell wall recalcitrance, developing genetic strategies to ease cell wall disintegration in pretreatment and fractionation processes and evaluation of novel techniques and methods for analysing cell wall recalcitrance.

Many of the genetic bases for the synthesis of cell walls have been identified in recent years. Still, some gaps remain. Especially, when it comes to the genetic connection with complex plant phenotypical traits such as the recalcitrance towards a specific degradation or destruction mode. Guo et al. identified the role of *Brittle culm 3* in barley, which encodes for a cellulose synthase subunit 5, and is required for cell wall biosynthesis. The brittle culm mutant was obtained by EMS mutagenesis and exhibited reduced mechanical strength of the culms due to impaired thickening of the sclerenchyma cell wall and reduced cellulose and hemicellulose content in the culms. A point mutation in the HvCESA5 gene predominantly expressed in the culms is responsible for the phenotype.

Most studies on genetic or biochemical traits are carried out under very controlled conditions. Transferring these results to the fields is necessary to develop strategies for an industrial application. Derba-Maceluch et al. investigated the impact of xylan on field productivity and wood saccharification properties in aspen. Their field trials included aspen trees with RNAi-induced reduction of xylan levels. The GALT1.1 and ASPR1 lines exhibited a skewed hemicellulose size distribution towards a higher molecular mass. Their findings indicated that GATL1.1 might function in xylan biosynthesis and that ASPR1 may regulate this process. Saccharification without pretreatment of these lines provided 8-11% higher average glucose yields if tested with additional acid pretreatment, the GT43BC construct provided a 10% yield increase on average. The field evaluation of transgenic xylan-reduced aspen represents an important step towards more productive feedstocks since it identified suitable (robust) target genes. Eudes et al. tested the performance of engineered switchgrass plants in field experiments. Overexpression of OsAT10, encoding a rice BAHD acyltransferase and QsuB, encoding dehydroshikimate dehydratase from *Corynebacterium glutamicum* enhanced the saccharification efficiency in switchgrass. But while showing chemotypic differences and reduced recalcitrance in greenhouse studies, the observed phenotype vanished when these plants were grown in field trials except for lightly increased biomass saccharification in QsuB lines.

The growing demand for plant-based materials within the transformation towards a sustainable bioeconomy poses challenges for the agricultural production sectors. Therefore, alternative concepts, e.g. producing biomass on marginal soils using specially adapted plant species and the improvement of existing crops for their utilization as raw material are important steps to overcome these challenges. Schrey et al. investigated the cell wall chemotype and saccharification yield of different genetically distant *Sida hermaphrodita* accessions. Sida is a non-domesticated species and their study aimed at expanding the potential of such plants in terms of their processability using genetically different accessions. American and European accessions showed contrasting cell wall characteristics and a different yield profile in OrganoCat pretreatment and fractionation experiments.

Wang et al. screened maize mutants with altered lignocellulosic properties. These chemically mutagenized plants, termed candy-leaf (cal) exhibited differences in their lignocellulosic composition but no obvious plant growth or developmental defects and have also an altered saccharification potential. These identified cal mutants are a valuable tool to decipher grass-specific aspects of cell wall recalcitrance and its genetic basis, by identification of responsible genes via the location of the mutation.

For the production of lignocellulose as a raw material, stable yields, and consistent or predictable quality are important. In this respect, studies that deal with the effects of environmental influences such as different nutrient and water supply or the effect of environmental stresses are very important. Jerbi et al. investigated the effect of primary wastewater irrigation on the wood structure and composition of the willow cultivar *Salix miyabeana* ‘SX67’ following two years of growth. The wastewater irrigation significantly altered the chemical composition of woody tissue and increased the glucan content and proportion of tension wood. Blervacq et al. showed the effect of gravitropic stress on flax plants by mapping the changes in organization and relative quantities of cell wall polymers in bast fibre cell walls by Raman spectroscopy. Their results showed that gravitropic stress induced discrete but significant changes in the composition and/or spatial organization of cellulose, hemicelluloses, and lignin within the cell walls of both the pulling side and the opposite side of the fibres.

Overall, the Research Topic provides insight into the latest findings and developments on understanding and overcoming plant cell wall recalcitrance. The presented research results addressed this challenge at different levels of its complexity and placed it in the broader context of biomass cultivation and utilisation towards platform chemicals, materials, and fuels.

## Author contributions

HK: Conceptualization, Writing – original draft, Writing – review & editing. GP: Conceptualization, Writing – original draft, Writing – review & editing.
